# Prebiotic Chemistry of Phosphite: Mild Thermal Routes to Form Condensed-P Energy Currency Molecules Leading Up to the Formation of Organophosphorus Compounds

**DOI:** 10.3390/life13040920

**Published:** 2023-03-31

**Authors:** Maheen Gull, Tian Feng, Harold A. Cruz, Ramanarayanan Krishnamurthy, Matthew A. Pasek

**Affiliations:** 1 School of Geosciences, University of South Florida, Tampa, FL 33584, USA; 2 Department of Chemistry, The Scripps Research Institute, La Jolla, CA 92037, USA

**Keywords:** phosphite, phosphorus, organophosphorus compounds, origin of life, condensed phosphates, condensation, phosphorylation, wet–dry cycles

## Abstract

The in-fall of meteorites and interstellar dust particles during the Hadean–Archean heavy bombardment may have provided the early Earth with various reduced oxidation state phosphorus compounds and minerals, including phosphite (HPO_3_^2−^)([Pi(III)]). The ion phosphite ([Pi(III)])has been postulated to be ubiquitous on the early Earth and consequently could have played a role in the emergence of organophosphorus compounds and other prebiotically relevant P species such as condensed P compounds, e.g., pyrophosphite ([PPi(III)]) and isohypophosphate ([PPi(III–V)]). In the present study, we show that phosphite ([Pi(III)]) oxidizes under mild heating conditions (e.g., wet–dry cycles and a prebiotic scenario mimicking a mildly hot-evaporating/drying pool on the early Earth at 78–83 °C) in the presence of urea and other additives, resulting in changes to orthophosphate ([Pi(V)]) alongside the formation of reactive condensed P compounds (e.g., pyrophosphite ([PPi(III)]) and isohypophosphate ([PPi(III–V)])) through a one-pot mechanism. Additionally, we also show that phosphite ([Pi(III)]) and the condensed P compounds readily react with organics (nucleosides and organic alcohol) to form organophosphorus compounds.

## 1. Introduction

Phosphorus (P, hereafter) is a key biologic element that is ubiquitous in biochemistry because phosphorylated biomolecules play central roles in many life-sustaining processes such as replication and information (as an essential component in RNA and DNA), in metabolism (as ATP and NADPH, etc.), and cellular structure (as phospholipids) [1]. It exists in various inorganic chemical forms including orthophosphate ([Pi(V)]), pyrophosphate ([PPi(V)]), triphosphate ([PPPi(V)]), phosphite ([Pi(III)]), phosphine ([Pi(III)]), and hypophosphite ([Pi(I)]). Living organisms use these various forms of inorganic P for the formation of organophosphorus compounds with C-O-P and C-P type linkages by utilizing various enzymes [1]. Orthophosphates ([Pi(V)]) (mainly in the form of minerals) are considered to be the major carriers of P on the surface of the Earth [1,2].

The geochemistry of P on the Hadean Earth may have been significantly altered by the meteoritic mineral schreibersite (Fe,Ni)_3_P ([Pi(0)]), which is believed to have been supplied by meteorites during the heavy bombardment period on the early Earth [3,4,5]. This mineral is found in many types of meteorites and in interplanetary dust particles [6] and readily reacts and corrodes into water to give inorganic P species such as phosphate ([Pi(V)]), condensed phosphates, and reduced oxidation state P compounds (simply called reduced P, hereafter), including phosphite ([Pi(III)]) and even hypophosphite ([Pi(I)]) [7,8]. This extraterrestrial mineral would have supplied a significant amount of P to the early Earth [9,10]. The total mass of reduced P that the late accretion period could possibly have delivered to Earth from 4.50 Ga to 3.50 Ga is estimated to be around 1.32 × 10^19^ kg [9].

In addition, for high-velocity impacts of large extraterrestrial objects (>100 m in diameter), the projectile partially evaporates and is distributed to the surroundings as fine-grained particles [1,11]. During the heavy bombardment period, the whole Earth could have been covered by mafic and metallic particles, both extremely reducing in nature relative to the surface of the Earth [12]. Furthermore, the vapor plume of the material resulting from an impact is also postulated to be reducing in nature and could potentially reduce phosphates present in the target material to their reduced form as phosphides [1]. The evidence to support this phenomenon is the detection of vast amounts of P as schreibersite in Lunar melts [13]. This indicates that a substantial amount of P delivered during meteoritic impacts was in a reduced form and also that the impact process itself could also have reduced phosphates to phosphides [14].

The possibility of such reduced P compounds being relevant to early Earth is further supported by the occurrence of phosphonic acids in the Murchison meteorite [15] and phosphite in ancient Archean marine carbonates [16], in natural glasses called fulgurites [17], in hydrothermal systems [18], in natural waters [19], and by the geochemical reduction of phosphates into phosphite [20].

Addison Gulick was the first to propose that reduced P compounds such as hypophosphite ([Pi(I)]) and phosphite ([Pi(III)]) could plausibly have been more relevant to the origin of life on the early Earth than phosphates [21]. These reduced P compounds tend to be around 10^3^ to 10^6^ times more soluble in water as compared to orthophosphate in the presence of certain common divalent cations [22,23]. The reduced P compounds are released from the aqueous corrosion of schreibersite and can react with organic compounds to form C-O-P and C-P type compounds, thus establishing this mineral as highly relevant to the prebiotic chemistry and the origin of life [6,7,8,16,24,25].

Recent studies have shown that despite its reactivity, phosphite ([Pi(III)]) can be stable enough to be detected in various natural environments [26]. It is therefore highly likely that this reduced P compound would have played an important role in the prebiotic P chemistry. Kee and colleagues suggested that phosphite ([Pi(III)]) would have played a significant role in the formation of reactive condensed inorganic P compounds including pyrophosphite ([PPi(III)]), isohypophosphate ([PPi(III–V)]), and pyrophosphate ([PPi(V)]), with the more energetically accessible pyrophosphite ([PPi(III)]) enabling the formation of pyrophosphate ([PPi(V)]) via an isohypophosphate ([PPi(III–V)]) intermediary [27]. Condensed P compounds, including pyrophosphate ([PPi(V)]), play an important role in biochemistry.

In the present study, starting with phosphite species, we report the formation of pyrophosphite ([PPi(III)]) in the presence of urea, various salts, and other additives such as minerals/clays, as potentially plausible condensation agents under mild conditions (78–83 °C, 1 atm) and through wet–dry cycles. In some reactions where phosphite ([Pi(III)]) condensed, we also observed the formation of inorganic phosphate possibly from the auto-oxidation of phosphite ([Pi(III)]) during heating through the wet–dry cycles or heating leading to dryness, which was primarily facilitated by the presence of urea. Besides urea, we also studied various other additives to test their potential roles in the formation of various prebiotically relevant P compounds. The additives included prebiotically relevant cations, e.g., Ca^2+^, Mg^2+^ [28,29,30], Na^+^ [31], NH_4_^+^ [32,33], CO_3_^2-^ [34], clays and other minerals [35,36], and urea [37,38]. We also show that the reaction mixture containing phosphite and the condensed P species readily reacts with nucleosides and organic alcohols to form organophosphites.

## 2. Materials and Methods

Sodium hypophosphite hydrate (NaH_2_PO_2_·H_2_O, 98%), phosphorous acid (H_3_PO_3_, 98%), sodium phosphite dibasic pentahydrate (Na_2_HPO_3_·5H_2_O), adenosine (C_10_H_13_N_5_O_4_, 98%), and deuterium oxide (D_2_O, 99.8% atom % D) were from Acros Organic; Uridine (C_9_H_12_N_2_O_6,_ 98%), standard compounds, e.g., uridine-5-monophosphate (5′-UMP) and adenosine-5-monophosphate (5′-AMP) were from Sigma Aldrich, urea, thiourea, kaolinite clay, calcium sulphate dihydrate (CaSO_4_·2H_2_O), magnesium chloride, sodium chloride, ammonium carbonate, ammonium chloride from TCI, calcium chloride (CaCl_2_, 98%), white sand (SiO_2_) and ferrous chloride tetrahydrate (FeCl_2_·4H_2_O, 98%), and instant ocean were from Alfa Aesar. Deionized water was obtained in-house using a Barnstead (Dubuque, IA, USA) NANO pure^®^ Diamond Analytical combined reverse osmosis-deionization system [24,25,26].

### 2.1. Synthesis of Inorganic Condensed P Compounds through Wet–Dry Cycles

0.100–0.150 g of P source (Table 1) was added to a clean glass vial (20 mL capacity) containing 7 mL DDI water (doubly deionized or ultrapure water). In some reaction samples, various additives were also added (Table 1) to test their plausible role as condensation agents for the formation of condensed P compounds. The contents were mixed and the initial pH was noted using pH paper. A small magnetic stirrer was added to the solution. The sample was allowed to heat on a hot plate, uncapped at 78–83 °C. After 24 h, the heat-dried mixture was rehydrated with 7 mL DDI water. The rehydrated sample was heated and after the completion of 48 h, it was rehydrated once again with 7 mL DDI water and heated, leading to a complete dryness. The reaction was stopped at exactly 72 h.

One reaction was also performed to specifically compare the possible role of urea in promoting the condensation reactions of phosphite ([Pi(III)]). In this reaction sample, instead of urea, thiourea was added to determine if it also promotes the heat driven oxidation of phosphite to phosphate. The reaction conditions were similar and the only difference was that instead of using urea, thiourea was added as an additive (Table 1, also see SI).

### 2.2. Synthesis of Inorganic Condensed P under ‘Warm-Pool Model’ Theme

This study was carried out to investigate the formation of condensed P compounds in a prebiotic scenario mimicking a mildly hot, evaporating/drying pool on the early Earth, as previously described [39]. 0.1 g of P source; hypophosphite (NaH_2_PO_2_·H_2_O) (Sample P1-U) or phosphite (Na_2_HPO_3_ 5H_2_O) and 0.5 g urea (Sample P3-NWD) (Table 1) were added to a clean glass vial (20 mL capacity) containing 7 mL DDI water. The contents were mixed and the initial pH (around 8.5) was noted using pH paper. A small magnetic stirrer was added to the solution. The sample was allowed to heat on a hot plate, uncapped at 78–83 °C for 2 days. After 2 days, the heat-dried mixture was removed from heating and was prepared to be analyzed by ^31^P-NMR.

### 2.3. Synthesis of Organophosphites from the Reactive Condensed P through Wet–Dry Cycles

0.1 g P source (sodium phosphite), 0.6 g–0.8 g organic compound (either a nucleoside: uridine or adenosine or an organic alcohol: 0.8 g glycerol), and 0.5 g urea were added to a clean glass vial of 20 mL capacity containing 7 mL of DDI water. The pH of the reaction mixture solution was 8.5. This solution was stirred using a small magnetic stirrer and was heated at 70–78 °C, uncapped for 24 h, to complete dryness. After 24 h, the dried reaction mixture was rehydrated with 7 mL DDI water. This reaction mixture was heated (uncapped) for another 24 h, after which the heat-dried reaction was stopped. In another set of experiments, identical reactions were carried out omitting urea (Table 2).

### 2.4. Analyses and Characterization of Inorganic and Organic P Compounds

For ^31^P-NMR analyses, the samples were analyzed on a 400-MHz Varian Unity Inova NMR operating at 161.9 MHz in both H-coupled and H-decoupled modes. The width of the spectrum was 200 ppm, and the running temperature was 22 °C. Various P products e.g., both inorganic and organic P compounds were quantified by peak integration method as previously reported [16,24,25,26,39,40,41].

The specific details of the ^31^P-NMR instrument and its related parameters have already been reported in our previous studies [16,23,24,25,26,39,40,41]. Each sample, completely dried out from heating, was cooled down to room temperature and was rehydrated with 5 mL DDI water. The reaction sample was mixed and stirred until a suspension was formed, which was filtered and centrifuged. The contents (2 mL) were then transferred to a clean watch glass followed by air-drying at room temperature. The air-dried room temperature sample was then rehydrated with a 2 mL D_2_O (90%) and DDI water (10%) solution (or only DDI water in the case of analysis for MS) and was centrifuged once again. The total volume of the solution was 2 mL. About 400 µL of the sample solution was transferred to a clean NMR tube and was analyzed by ^31^P-NMR.

Mass spectrometry (MS) analyses were formed in negative ion mode on a 6130 Single Quadrupole Mass Spectrometer (Agilent, Santa Clara, CA, USA) attached to an Agilent 1200 HPLC by direct injection, and deionized water was used as a solvent as reported previously [16,40,41].

Organophosphorus compounds including 5′-AMP and 5′-UMP were confirmed by spiking with the standard compounds as previously [33] (see also SI). Remaining organophosphorus compounds including organic phosphites were identified and characterized by studying their characteristic peak splitting in the H-coupled ^31^P-NMR, measuring their *J* coupling constants, and finding the target peaks in the mass spectrometer.

## 3. Results

**Figure 1:**
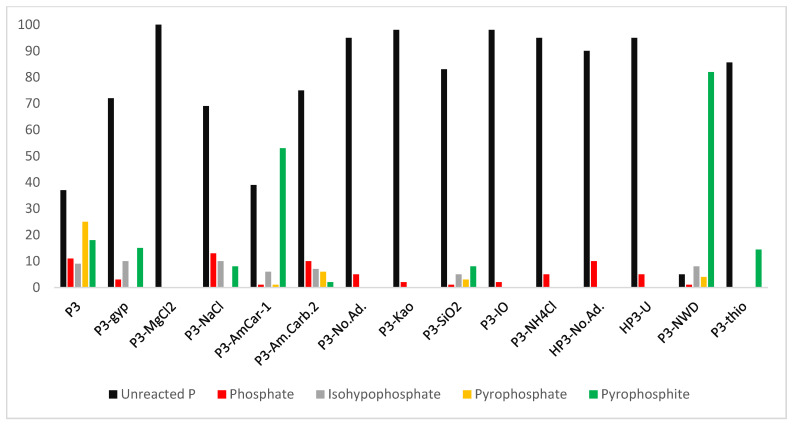
Yields (abundances (%)) of various condensed P compounds in various samples. Where unreacted P means the starting P source, which in this case is either phosphite or phosphorous acid. The *Y*-axis represents the abundance (%), whereas the *X*-axis represents the various samples.

Heating inorganic P compounds through wet–dry cycles at 78–83 °C resulted in the formation of condensed P species. The reaction samples produced high-energy condensed P compounds that reacted with organic substrates (Table 3, Figure 1, see also SI, Figures S1 and S2). When sodium phosphite ([Pi(III)]) was heated in the presence of urea, pyrophosphite [PPi(III)] was generated. In some samples of the above-mentioned reactions, to our surprise, we also detected orthophosphate [Pi(V)]. To further confirm the presence and source of orthophosphate [Pi(V)], ^31^P-NMR of blank sodium phosphite [Pi(III)] did not show any presence of orthophosphate [Pi(V)] (SI, Figure S2). This confirmed that phosphite under mild heating conditions and through the wet–dry cycling has a tendency to autoxidize to orthophosphate [Pi(V)]. Subsequently, this orthophosphate [Pi(V)] reacts with pyrophosphite [PPi(III)] to form a mixed valence condensed P species isohypophosphate [PPi(III–V)] [27].

**Figure 2:**
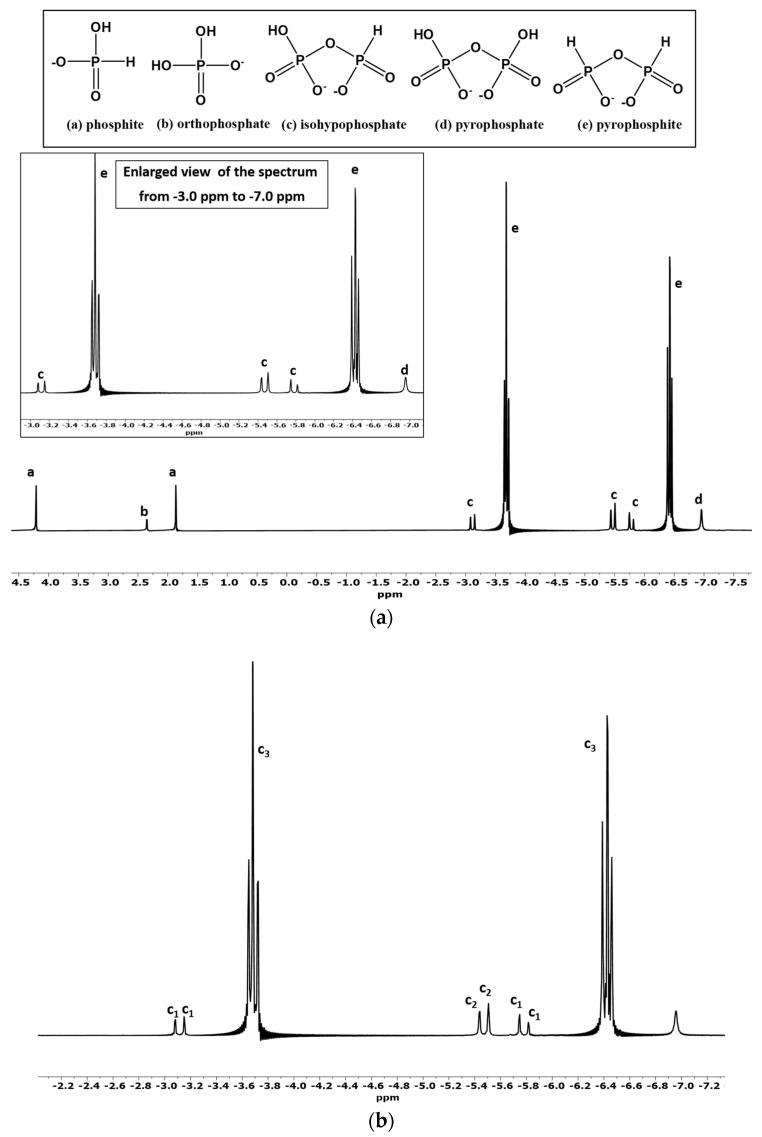
(**a**)**.** H-coupled ^31^P-NMR of condensation (and auto-oxidation) reaction of phosphite [Pi(III)] in the presence of urea (Sample P3-NWD). The labeled peaks represent the following compounds: (a) phosphite [PPi(III)], (b) orthophosphate [Pi(V)], (c) isohypophosphate [PPi(III–V)], (d) pyrophosphate [PPi(V)], and (e) pyrophosphite [PPi(III)]; (**b**) enlarged spectrum from −2.0 ppm to −7.2 ppm showing various coupling constants: C1 and C2 (coupling constants (ẟ values) show the presence of isohypophosphate, and C3 represents the coupling constants values for pyrophosphite [PPi(III)].

H-coupled ^31^P-NMR analysis confirmed isohypophosphate [PPi(III–V)] in the form of three doublets in the −3.0 to −6.0 ppm region, as reported previously [27]. The peaks in our results were slightly shifted from the previously reported values: −2.5 to −7.0 ppm [27], to −3.0 to −6.0 ppm. This slight shift in the location of the peak and chemical shift values was attributed to the pH changes [42]. Peak c (isohypophosphate) [PPi(III–V)] was identified by the following coupling constant values [27]: δ–4.44 [dd, c_1_J_PH_ = 645 Hz, c_1_J_PP_ = 17.36 Hz, Pi(III)]; δ–5.50 [d, c_2_J_PP_ = 17.22 Hz, Pi(V)]. These values are within the range as reported previously [27]. Pyrophosphite [Ppi(III)] was identified as two triplets in the H-coupled mode of ^31^P-NMR; one triplet around −3.6ppm and the other around −6.5 ppm, as also reported previously [27]. Peaks e (pyrophosphite) at δ–5.05 [dd, c_3_ J_PH_ = 660 Hz, PPi (III)] correspond to phosphite triplet splitting (Figure 2a,b).

Pyrophosphate [PPi(V)] was identified as a singlet in H-coupled mode of ^31^P-NMR around the −7 ppm region. Figure 2 shows the ^31^P-NMR spectrum of the reaction sample containing phosphite, urea, and heating leading to dryness at 78–83 °C for 3–4 days (without wet–dry cycles).

In order to confirm that heating leading to dryness and wet–dry cycles accompanied with heating can result in the auto-oxidation of phosphite [Pi(III)], we also studied the similar reactions of sodium hypophosphite [Pi(I)], representing another source of reduced P. When the latter was heated leading to dryness for 3–4 days at 78–83 °C, we observed phosphite [Pi(III)] and even phosphate [Pi(V)], implying that the orthophosphate [Pi(V)] was not an impurity but was actually a product of auto-oxidation of a reduced P compound (SI, Figures S3 and S4). We also analyzed (^31^P-NMR), the solutional blanks containing 1 pure hypophosphite [Pi(I)] (SI, Figures S2 and S4) phosphite [Pi(III)] (SI, Figure S2). The ^31^P-NMR analysis did not reveal any peaks containing phosphate [Pi(V)], suggesting the reduced P compounds did undergo auto-oxidation (SI) on heating through wet–dry cycles or heating leading to dryness.

Heating phosphite [Pi(III)] via wet–dry cycles at 78–83 °C in the presence of urea produced condensed P compounds up to 60% (% abundance) (Table 1, Sample P3-Am.Carb.1) and 52% in the case of Sample P3 (Table 1, Sample P3). Sodium carbonate (along with urea) seemed to promote the condensation of phosphite (Table 1, Sample P3-Am.Carb.1; see also Supplementary information (SI)). However, when the concentration of ammonium carbonate was doubled, it significantly declined the rate of condensation of phosphite [Pi(III)]. The other additives including salts, instant ocean (IO), minerals, and clays did not seem to positively enhance the condensation process of phosphite. Urea seemed to be an excellent additive (and condensation agent) in all the different reactions attempted. The highest production of condensed P products (pyrophosphite [PPi(III)], pyrophosphate [PPi(V)], and isohypophosphate [PPi(III–V)]) was obtained by the simple heating of the solution mixtures of phosphite [Pi(III)] and urea to dryness without wet–dry cyclic treatment with yields (% abundances) of up to 94% (Figure 2, Sample P3-NWD, Table 3). Overall, the condensation reactions required urea and heating the solutions at 78–83 °C for 3–4 days and proceeded smoothly following either route i.e., wet–dry cycles or heating leading to dryness.

Both starting reduced P compounds (hypophosphite [Pi(I)] and phosphite [Pi(III)]) seemed to undergo auto-oxidation (see also SI, Figures S2–S4). This auto-oxidation of phosphite [Pi(III)] produced phosphate, whereas the auto-oxidation of hypophosphite [Pi(I)] produced phosphite [Pi(III)] and phosphate [Pi(V)]. These reduced P (hypophosphite [Pi(I)] and phosphite [Pi(III)]) compounds at the same time also condensed to form various condensed P compounds, including mixed-valence state P compounds such as isohypophosphate [PPi(III–V)].

Furthermore, the reactions seemed to be pH sensitive. The best yields were obtained around pH = 8, whereas at lower pH (in case of H_3_PO_3_, pH = 2), we did not observe any condensed P compounds, though heating and the wet–dry cycle promoted the auto-oxidation of H_3_PO_3_ to form orthophosphate [Pi(V)] (Table 1 reaction Samples HP3-No.Ad. and HP3-U). As mentioned above, when reaction samples (solutions) were heated to a complete dryness at 78–83°C for two days without wet–dry cycles, the relative abundances (% yields) of the condensed compounds were around 94%, with pyrophosphite [PPi(III)] yield being around 82%. This particular reaction gave the best possible yields of the condensed P compounds but the observed yield of orthophosphate [Pi(V)] (oxidation product of phosphite [Pi(III)]) was only 1% (Table 3, reaction Sample P3-NWD, Figure 2). The other samples with better yields (% abundances) of inorganic condensed P species including pyrophosphite [PPi(III)], pyrophosphate [PPi(V)], and isohypophosphate were seen when phosphite [Pi(III)] was heated in the presence of urea and heated through wet–dry cycles (Table 3). Heating leading to dryness, therefore, favored the formation of pyrophosphite [PPi(III)] over other condensed P compounds. The rationale behind would be that the formation of pyrophosphite [PPi(III)] occurs quicker than the multistep conversion of isohyphopshate [PPi(III–V)], for (1) oxidation of phosphite [Pi(III)], to orthophosphate [Pi(V)], (2) condensation of phosphite [Pi(III)], with phosphate [Pi(V)], to form isohypophopshate [PPi(III–V)], (3) condensation of orthophosphate [Pi(V)], to form pyrophosphate [PPi(V)].

Urea seemed to promote the condensation as well as oxidation reactions of the reduced P compounds. Although, the auto-oxidation of the reduced P compounds could be promoted without the urea (Table 2 and Table 3, Sample P3-No.Ad.), higher (relative abundances) yields of phosphate [Pi(V)] were observed when urea was present. The supportive role of urea was further explored when thiourea was used in place of urea. In case of thiourea as an additive, no phosphate [Pi(V)] was observed (it was below detection limits) and pyrophosphite [PPi(III)] was the only product observed, suggesting that thiourea did promote the condensation but not the oxidation of phosphite [Pi(III)] (SI, Figure S7).

**Figure 3:**
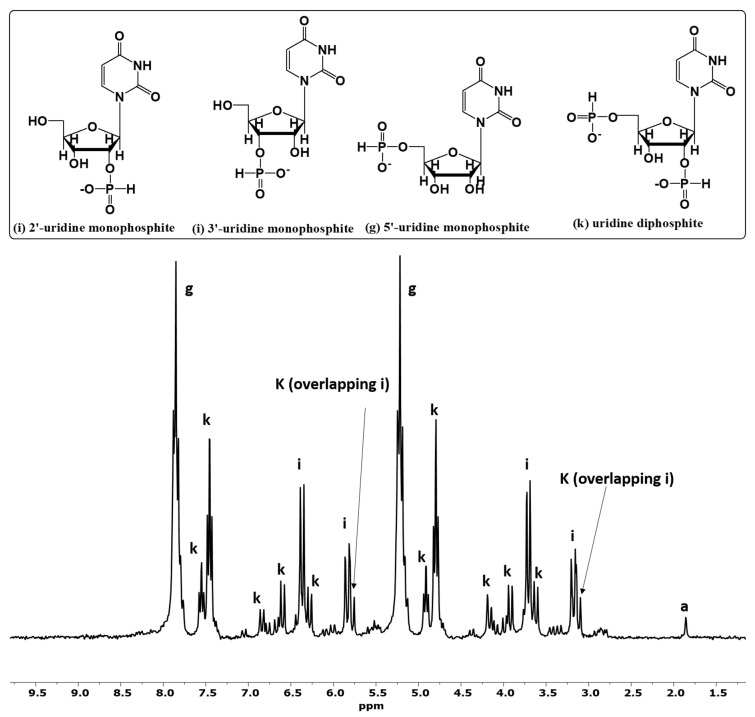
H-coupled ^31^P-NMR of phosphorylation and phosphonylation reactions of uridine (sample B). The labeled peaks correspond to the following compounds: (a) phosphite, (g) 5′-uridine phosphite, (i) 2′and 3′-uridine phosphites, and (k) various diphosphite (not pyrophosphite) species of uridine. Various isomers are possible in this case, e.g., uridine 2,3-diphosphite, uridine 2,5-diphosphite, etc. Each isomer in the H-coupled mode of ^31^P-NMR splits into two triplets and two doublets (peaks k). Such diphosphite species were identified by distinctive peak splitting patterns i.e., doublet of doublets and doublet of triplets indicated the presence of various organic diphosphite species.

In order to further investigate the reactivity of these high-energy condensed P compounds, we also studied the phosphonylation reaction of nucleosides (adenosine and uridine) and organic alcohol (glycerol) with the reaction mixtures containing phosphite [Pi(III)] and urea (Table 2). The reaction mixture readily reacted with an organic compound in the presence of urea at 70–80 °C through wet–dry cycles and produced organic phosphites as expected. However, some organophosphates were also observed. The organophosphates were observed possibly due to the reaction between the orthophosphate [Pi(V)] formed as a consequence of the auto-oxidation of phosphite [Pi(III)] and a nucleoside. In the case of organic alcohol (glycerol), we did not observe any organophosphates, which implied that the rate of phosphonylation of glycerol was faster than the auto-oxidation of phosphite [Pi(III)] (Figure 3).

The presence of various organophosphorus compounds was confirmed by ^31^P-NMR peak characterization as well as by MS (the direct injection method), as reported previously [33,39,40,41]. The direct injection MS of reaction sample containing glycerol showed the following major peaks: [C_3_H_9_O_5_P-H] at *m*/*z* 155.02 corresponding to glycerol phosphite and [C_3_H_10_O_7_P_2_-H] at *m*/*z*: 218.99 corresponding to glycerol diphosphite. In the reaction samples with uridine, we observed: [C_9_N_2_O_6_H_11_-H] at *m*/*z* 243 corresponding to uridine nucleoside, [C_9_N_2_O_9_PH_13_-H] at *m*/*z* 323.04 corresponding to uridine-monophosphate (2′, 3′ and 5′-UMP species), and [C_9_N_2_O_8_PH_12_-H] at *m*/*z* 307 corresponding to uridine-monophosphite. Similarly, the major peaks in MS were identified for the adenosine reaction in the reaction samples. For the solutions with adenosine, we observed: [C_10_H_13_N_5_O_4_-H] at *m*/*z* 266 corresponding to adenosine nucleoside, [C_10_H_13_N_5_O_7_P-H] at m/z 346 corresponding to monophosphate (2′, 3′ and 5′-AMP species), and, finally, [C_10_H_14_N_5_O_6_P-H] at *m*/*z* 330 corresponding to adenosine-monophosphite.

The formation of the organophosphorus compounds was improved by the presence of urea (Table 4). Heating uridine with an aqueous solution of phosphite [Pi(III)] and urea produced various uridine phosphites. Figure 3 (Sample B) shows ^31^P-NMR analysis of Sample B in H-coupled mode. Various uridine phosphite species were identified and characterized by observing their chemical shift values; C-O-P (carbon, oxygen and phosphorus) and P-H interactions [43]. Figure 3 shows the various P species, including both organic and inorganic P compounds, and is without any spiking with the standard compounds. In the reaction sample B, we did not observe any organophosphates. However, various species of uridine phosphites were observed. The organophosphite 5′-uridine phosphite (peak g) was identified in the form of two triplets around 5.2 and 7.8 ppm; 2′- and 3′-uridine phosphites (peaks i) were identified as two doublets. Various other doublets and triplets labeled as peaks k represent uridine diphosphite species. These uridine diphosphite species (peaks k) represent uridine-P species having one phosphite group attached to 5′- position and another either to 2′- or to 3′-positions but not through a pyrophospite [PPi(III)] (P-O-P) type linkage. Each triplet and a doublet, in the case of phosphite, [Pi(III)] splits further into another triplet and a doublet, representing uridine-diphosphite species. The total yield (% abundance) of organophosphite in the case of uridine was 99%. When phosphite [Pi(III)] was heated without urea (Sample A), the yields (% abundances) of the uridine phosphates and phosphites were lower. These yields represent the relative abundances (%) of the phosphonylated/phosphorylated products and were calculated on the basis of the total P dissolved and by the peak integration method as reported before [24,25,26].

**Figure 4:**
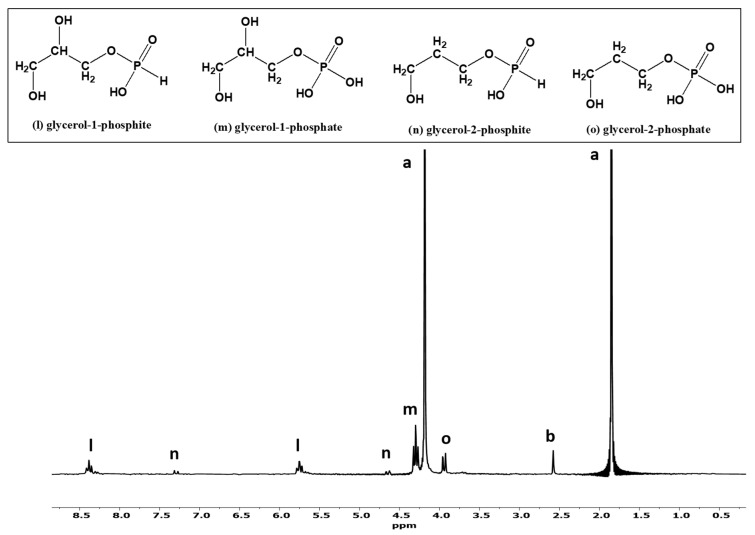
H-coupled ^31^P-NMR of phosphorylation and phosphonylation reactions of glycerol (sample D). The labeled peaks correspond to the following P compounds: (a) phosphite, (b) phosphate, (l) glycerol-1-phosphite, (m) glycerol-1-phosphate, (n) glycerol-2-phosphite, and (o) glycerol-2-phosphate. The figure also shows the enlarged peaks from 2.8 to 8.4 ppm.

In the glycerol and phosphite sample without urea (Sample C), only 5% glycerol phosphites were detected by ^31^P-NMR, whereas for the other sample containing glycerol, phosphite [Pi(III)], and urea, the yield (relative abundances) of the glycerol phosphites reached around 20% (reaction Sample D, Figure 4). Figure 4 shows ^31^P-NMR analysis of Sample D in H-coupled mode. Again, various glycerol phosphite species were identified and characterized by observing their chemical shift values; C-O-P (carbon, oxygen and phosphorus) and P-H interactions [42,43,44]. Glycerol-1-phosphite was identified by two triplets (peaks l) and glycerol-2-phosphite was confirmed by two doublets (peaks n). Similarly, triplet peak m represents glycerol-1-phosphate, and peak o (doublet) represents glycerol-2-phosphate (Figure 4).

**Figure 5:**
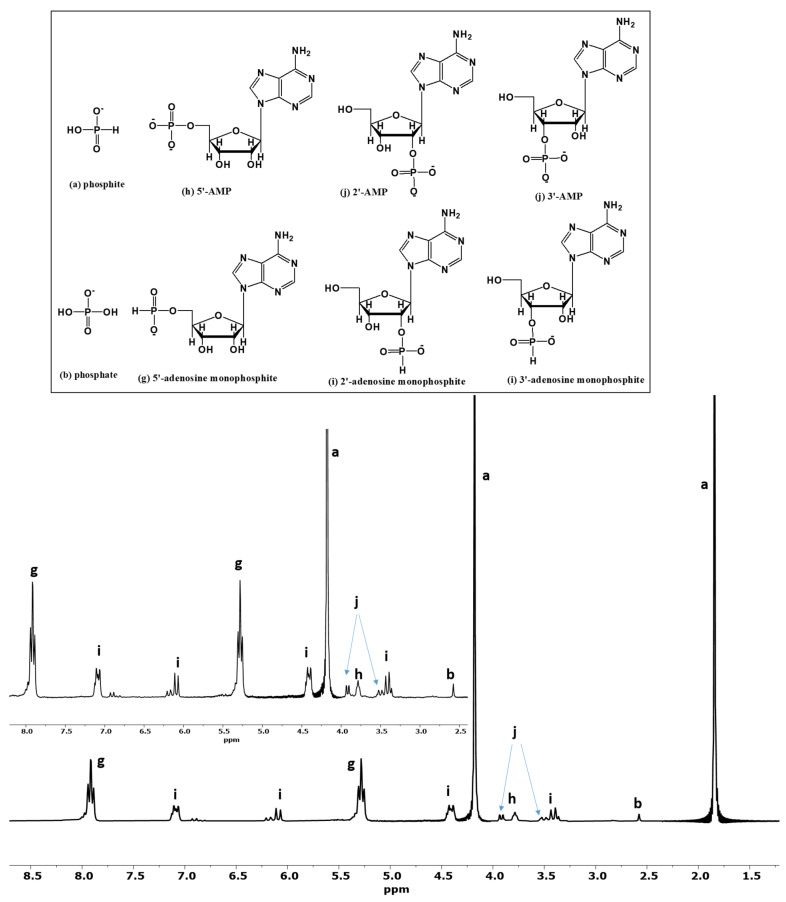
H-coupled ^31^P-NMR of phosphorylation and phosphonylation reactions of adenosine (Sample F). The labeled peaks represent the following compounds: (a) phosphite, (b) phosphate, (g) 5′-adenosine phosphite, (h) 5′-AMP, (i) 2′and 3′-adenosine phosphites, and (j) 2′and 3′-AMP.

**Figure 6:**
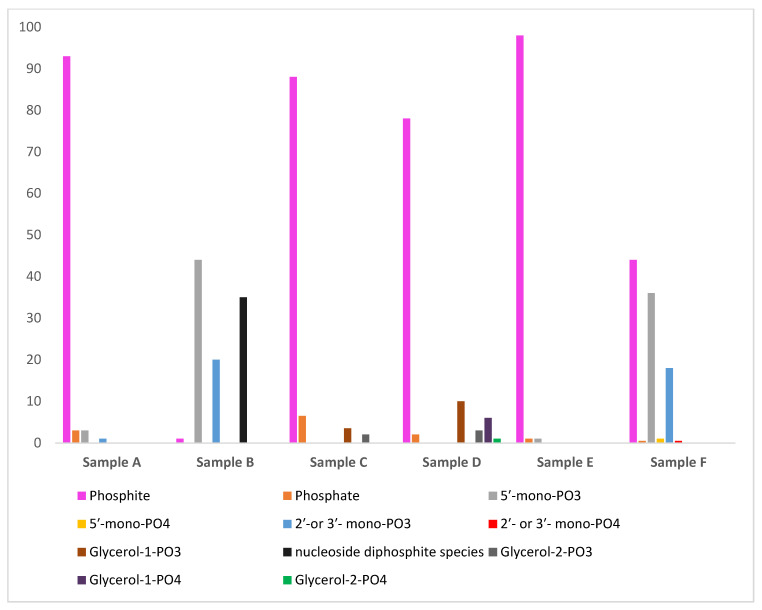
The relative abundances (%) of the phosphonylated and phosphorylated as well as amount of inorganic phosphate (oxidation product of phosphite) observed in each sample. Where Samples B, D, and F where with urea as an additive, and Samples A, C, and E were without urea. The relative abundances (%) were calculated using the peak integration method of ^31^P-NMR [24,25,26].

Similar reaction trends were seen in the case of adenosine nucleoside. On heating (and through wet–dry cycles) adenosine with the phosphite solution in the presence of urea, the yields of adenosine-P (both phosphates and phosphites) reached around 55.5%; this declined to only 1% when urea was not included. Urea played a significant role in the C-O-P bond formation. Various reaction products of adenosine-P are shown in Figure 5. Peak labeling and identification is consistent with that of Figure 4. Nucleotides including 5′-AMP and 5′-UMP, if any, present in each one of the respective reaction samples were also spiked with standard 5′-AMP and 5′-UMP solutions, as mentioned in Section 2.4 and as previously described [33] (Figure 6, see also SI, Figure S6a,b).

Organophosphites (phosphites of glycerol, uridine, and adenosine) were also identified by observing their coupling constants; for example, the coupling constant (d (delta) value for the two organophosphite doublets or triplets in the H-coupled ^31^P-NMR were around 640–655 Hz, a clear indication of the presence of phosphites derivatives of organics [43].

## 4. Discussion

Heating phosphite with urea at 78–83 °C with wet–dry cycles (or heating leading to dryness e.g., without wet–dry cycles ‘warm alkaline pool scenario’) lead to the oxidation of some of the phosphite [Pi(III)] to orthophosphate [Pi(V)] (1–13%), along with the formation of various condensed inorganic P compounds including pyrophosphate[Pi(V)], pyrophosphite [PPi(III)], and isohypophosphate [PPi(III–V)]. An interesting finding in the reaction system was the auto-oxidation of phosphite into phosphate [Pi(III)] and hypophosphite [Pi(I)] into phosphite [Pi(III)] and even phosphate. At present, we do not know the exact mechanism. Since phosphate [Pi(V)] was also detected in all samples, even the one without urea (Table 1, Sample Na-12), no additive seemed to be required to promote the auto-oxidation under the air of phosphite [PPi(III)] into phosphate [Pi(V)] and hypophosphite [Pi(I)] into phosphate [Pi(V)] and phosphite [Pi(III)]. A plausible explanation would be that evaporation and heating leading to dryness somehow causes this autooxidation of phosphite into phosphate [Pi(V)].

**Figure 7:**
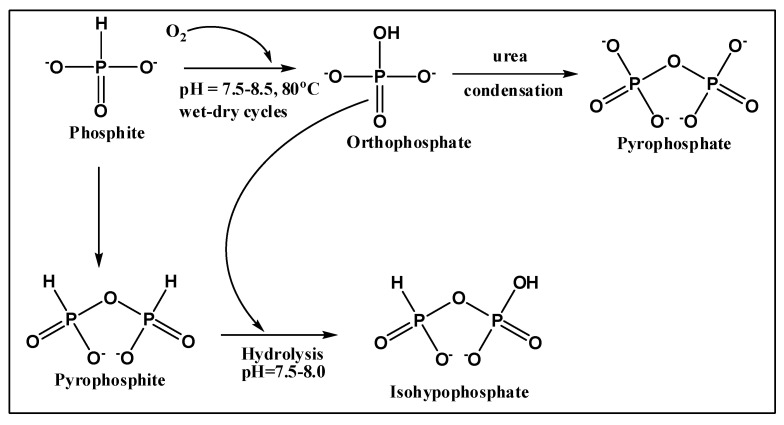
Description of various reaction steps suggested in the text.

In our studies, we made the following observations (Figure 7): (1) some amount of phosphite [Pi(III)] was oxidized to orthophosphate [Pi(V)] (1–13%) during the prolonged heating (3–4 days) at 78–83 °C through wet–dry cycles; (2) phosphite [Pi(III)] condensed in the presence of urea into pyrophosphite [PPi(III)]; (3) this pyrophosphite [PPi(III)] subsequently hydrolyzed into phosphite [Pi(III)] to react with the orthophosphate [Pi(V)] generated via self-oxidation of phosphite to form isohypophosphate [PPi(III–V)]; and (4) some of the orthophosphate [Pi(V)] also condensed in the presence of urea to form pyrophosphate [PPi(V)]. Although, these reaction steps are *one-pot*, it is not clear how isohypophosphate [PPi(III–V)] is being produced (e.g., either by the hydrolysis of pyrophosphite [PPi(III)], as reported previously [27], or the reactant (phosphite) [Pi(III)] reacting with the phosphate [Pi(V)] present in the solution to form this mixed valence state P compound [PPi(III–V)].

To better understand the mechanism, we also studied the heating (leading to complete dryness) reactions of hypophosphite [Pi(I)] at 78–83 °C in the presence of urea for 2 days. We observed the oxidation and condensation reactions of hypophosphite [Pi(I)]. In the oxidation process, hypophosphite [Pi(I)] was oxidized into phosphite [Pi(III)] and phosphate [Pi(V)]. The condensed products included pyrophosphite [PPi(III)], isohypophosphate [PPi(III–V)], and pyrophosphate [PPi(V)]. This showed that overall inorganic reduced P compounds have a tendency to oxidize in air. The mechanism of the reaction can be compared with previously reported work by Kee and colleagues [27]. As suggested, pyrophosphite [PPi(III)] reacts readily with aqueous solutions of orthophosphate [Pi(V)] to give isohypophosphate, PPi(III–V) [27].

Previous studies have shown that hypophosphite [Pi(I)] can be considered a plausible intermediate in oxidation state between phosphide [Pi(0)] and phosphite [Pi(III)] [8]. Moreover, monitoring hypophosphite [Pi(I)] over the course of one day showed that 25% of this compound was oxidized to phosphite under air, independent of other additives in the solution [8]. Phosphite [Pi(III)], however, is more stable than hypophosphite [Pi(I)] over longer periods of time, hence it can be found in ancient rock samples [16] or in meteoritic solutions preserved for years [26]. The stability and longevity of phosphite [Pi(III)] in any solution is mainly dependent on the amount of oxidizing radicals in solution and the metals bonded to phosphite. CaHPO_3_, for example, is significantly more resistant to oxidation than Na_2_HPO_3_. It can be stabilized under mildly reducing conditions that can potentially remove oxidants (oxidizing agents) from solutions [1].

Another study suggests a route of conversion of hypophosphite [Pi(I)] and phosphite [Pi(III)] through wet oxidation at 453 K (180 °C) under a partial oxygen pressure of 0.5–5 MPa [45]. The study found only a slight oxidation of hypophosphite [PPi(III)] under a N_2_ atmosphere. However, in the presence of an O_2_ atmosphere of 1 Mpa, hypophosphite [Pi(I)] was decreased to 5% after 180 min. Furthermore, for the wet-oxidation of phosphite [Pi(III)] under an O_2_ pressure of 0.5–5 Mpa, the oxidation of phosphite [Pi(III)] to phosphate [Ppi(V)] was increased with an increase in the partial pressure of O_2_. The oxidation of phosphite [Pi(III)] to phosphate [Pi(V)] was also increased when the pH was lowered from 6.05 to 1.04 [45]. We, however, did not observe any impact of pH on the oxidation of phosphite to phosphate during these studies. For example, in all samples containing sodium phosphite [Pi(III)] (pH = 8.5), phosphorous acid [Pi(III)] (pH = 2), and hypophosphite [Pi(I)] (pH = 4–5), around 1–13% phosphate [Pi(V)] was detected, which was not found consistent with any specific pH range.

Phosphite [Pi(III)] is considered to be thermodynamically unstable but kinetically stable on the Earth’s surface [1]. The rate-limiting step in the oxidation process of phosphite [Pi(III)] is considered to be the breaking of the P-H (phosphorus-hydrogen) bond, which has a large activation energy of around 370 kJ [1]. This P-H bond, however, can be broken via a radical exchange mechanism (i.e., the reaction of phosphite with an ^•^OH radical to form H_2_O and PO_3_^2−^). Further investigation into the free radical formation from phosphide [Pi(0), e.g., schreibersite] has shown that O_2_ from air does not participate in forming the radical species [8].

The yields (% abundances) of the organic-P compounds were remarkably improved when urea was used as an additive (Figure 6). Urea seemed to facilitate condensation to form high-energy inorganic P compounds that readily reacted with the organics in contrast to the sample solution containing phosphite and organic without urea. In our reactions, we did not observe any organo-pyrophosphite species (P-O-P). One possibility is the instability and quick hydrolysis of such compounds. These results are also comparable with our recent studies that show one-pot syntheses of organic phosphates and phosphites specifically favored under alkaline conditions and additives such as urea and NH_4_^+^ ions [33]. The idea that high-energy condensed phosphates (and in this case condensed phosphites) are formed in the presence of urea that readily reacts with organics is also supported by our previous studies [39]; these show that pyrophosphate reacts with uridine to form uridine monophosphates alongside dimer (e.g., uridine-phosphate-uridine) [39]. However, the reaction of ‘high-energy phosphorus compounds’ formed in the mixture with the organics has not been verified independently e.g., by taking out the high-energy condensed P compounds from the crude reaction mixture and reacting them with organic substrates. Recently, it has been shown that phosphorous acid yields around 32.6% 5′-nucleoside monophosphate, along with di- and tri- organophosphate species, in a single reaction step at room temperature using liquid SO_2_ under prebiotic conditions. Simultaneous oxidation results exist for the formation of organophosphates from phosphorus ([Pi(III)]) acid [46].

On the whole, it is quite plausible to envision cycles of de- and rehydration on the early Earth that could have driven nucleic acid polymerization in a volcanic environment enriched in phosphite and sulfur [47]. More research will be needed to find the likely pathway of the plausible oxidation of phosphite [Pi(III)] into phosphate [Pi(V)] under such mild conditions and the plausible formation of condensed P compounds that can readily react with organics to form a one-pot mixture of organic phosphates and phosphites.

## Figures and Tables

**Table 1:** Reaction conditions of various reaction samples for the synthesis of various inorganic condensed P compounds.

Sample	Description
P3	0.1 g Na_2_HPO_3_ 5H_2_O, 7 mL DDI water, 0.5 g urea, pH = 8.5
P3-gyp	0.1 g Na_2_HPO_3_ 5H_2_O, 7 mL DDI water, 0.5 g urea, 0.2 g CaSO_4_·2H_2_O, pH = 8.5
P3-MgCl_2_	0.1 g Na_2_HPO_3_ 5H_2_O, 7 mL DDI water, 0.5 g urea, 0.2 g MgCl_2_, pH = 8.5
P3-NaCl	0.1 g Na_2_HPO_3_ 5H_2_O, 7 mL DDI water, 0.5 g urea, 0.2 g NaCl, pH = 8.0
P3-Am.Carb.1	0.1 g Na_2_HPO_3_ 5H_2_O, 7 mL DDI water, 0.5 g urea, 0.4 g (NH_4_)_2_CO_3_, pH = 8.0
P3-Am.Carb.2	0.1 g Na_2_HPO_3_ 5H_2_O, 7 mL DDI water, 0.5 g urea, 0.8 g (NH_4_)_2_CO_3_, pH = 8.0
P3-No.Ad.	0.1 g Na_2_HPO_3_ 5H_2_O, 7 mL DDI water, no additive, pH = 7.5
P3-Kao.	0.1 g Na_2_HPO_3_ 5H_2_O, 7 mL DDI water, 0.5 g urea, 0.3 g kaolinite, pH = 8.5
P3-SiO_2_	0.1 g Na_2_HPO_3_ 5H_2_O, 7 mL DDI water, 0.5 g urea, 0.25 g white sand, pH = 8.5
P3-IO	0.1 g Na_2_HPO_3_ 5H_2_O, 7 mL DDI water, 0.5 g urea, 0.25 g instant ocean, pH = 8.0
P3-NH_4_Cl	0.1 g Na_2_HPO_3_ 5H_2_O, 7 mL DDI water, 0.5 g urea, 0.4 g NH_4_Cl, pH = 8.0
HP3-No.Ad.	0.1 g H_3_PO_3_, 7 mL DDI water, no additive, pH = 2
HP3-U	0.1 g H_3_PO_3_, 7 mL DDI water, 0.5 g urea, pH = 2
P3-NWD	0.1 g Na_2_HPO_3_ 5H_2_O, 7 mL DDI water, 0.5 g urea, pH = 8.5
P1-U	0.1 g NaH_2_PO_2_·H_2_O, 7 mL DDI water, 0.5 g urea, pH = 6.0, NWD
P3-thio.	0.1 g Na_2_HPO_3_ 5H_2_O, 7 mL DDI water, 0.5 g thiourea, pH = 8.5

Prebiotic synthesis of condensed-P compounds. Various conditions tried in the study. Each of the samples were heated uncovered at 78–83 °C for 3 days and were given wet–dry cycle treatment for 3 days e.g., every 24 h. Each sample received three wet and three dry cycles. The meanings of the abbreviations used are; P3 (phosphite), gyp (gypsum), Am.Carb.1 (ammonium carbonate), P3-No.Ad. (phosphite with no additive), Kao. (kaolinite), IO (instant ocean), HP3-No.Ad. (phosphorous acid with no additive), HP3-U (phosphorous acid with urea), P3-NWD (phosphite with no wet–dry cycles), P1 (hypophosphite), and thio means (thiourea). Samples P3-NWD and P1-U represent ‘Warm-Pool Model’ Theme suggested in Section 2.2, whereas all the other samples represent ‘wet–dry cycles’ scenario suggested in Section 2.1, respectively.

**Table 2:** Reaction conditions of various reaction samples to study phosphonylation of organic molecules.

Sample	Description
A	0.1 g Na_2_HPO_3_ 5H_2_O, 7 mL DDI water, 0.6 g uridine, pH = 8, 70–72 °C
B	0.1 g Na_2_HPO_3_ 5H_2_O, 7 mL DDI water, 0.5 g urea, 0.6 g uridine, pH = 8.5–9, 70–72 °C
C	0.1 g Na_2_HPO_3_ 5H_2_O, 7 mL DDI water, 0.8 g glycerol, pH = 7.5, 73–75 °C
D	0.1 g Na_2_HPO_3_ 5H_2_O, 7 mL DDI water, 0.5 g urea, 0.8 g glycerol, pH = 8.5–9, 73–75 °C
E	0.1 g Na_2_HPO_3_ 5H_2_O, 7 mL DDI water, 0.65 g adenosine, pH = 7.5, 78–80 °C
F	0.1 g Na_2_HPO_3_ 5H_2_O, 7 mL DDI water, 0.65 g adenosine, 0.5 g urea, pH = 8.5–9, 78–80 °C

Prebiotic synthesis of organophosphites. Various conditions used in the study. Each of the samples were heated uncovered at from 70–78 °C for 2 days and were given wet–dry cycle treatment for 2 days, e.g., every 24 h. Each sample, therefore, received two wet and two dry cycle.

**Table 3:** The relative abundances (%) of various inorganic P products produced in various reactions.

Sample	Unreacted P	Phosphate	Isohypophosphate	Pyrophosphate	Pyrophosphite	P_T_
P3	37	11	9	25	18	52
P3-gyp	72	3	10	BDL	15	25
P3-MgCl_2_	100	BDL	BDL	BDL	BDL	BDL
P3-NaCl	69	13	10	BDL	8	18
P3-Am.Carb.1	39	1	6	1	53	60
P3-Am.Carb.2	75	10	7	6	2	15
P3-No.Ad.	95	5	BDL	BDL	BDL	BDL
P3-Kao.	98	2	BDL	BDL	BDL	BDL
P3-SiO_2_	83	1	5	3	8	16
P3-IO	98	2	BDL	BDL	BDL	BDL
P3-NH_4_Cl	95	5	BDL	BDL	BDL	BDL
HP3-No.Ad.	90	10	BDL	BDL	BDL	BDL
HP3-U	95	5	BDL	BDL	BDL	BDL
P3-NWD	5	1	8	4	82	94
P3-thio.	85.5	0.1	BDL	BDL	14.4	14.4

The relative abundances (%) of the inorganic P products were calculated on the basis of the total P dissolved and by the peak integration method as previously reported [24,37,38,39]. Various P sources used in the samples include; Na_2_HPO_3_·5H_2_O, H_3_PO_3_, or NaH_2_PO_2_·H_2_O (Table 1). Furthermore, the amount (%) of orthophosphate detected was produced by the oxidation of phosphite. Some of the abbreviations meanings are as follows: BDL (below detection limit) and P_T_ (total inorganic condensed P compounds generated). The meanings of the abbreviations used are; P3 (phosphite), gyp (gypsum), Am.Carb.1 (ammonium carbonate), P3-No.Ad. (phosphite with no additive), Kao. (kaolinite), IO (instant ocean), HP3-No.Ad. (phosphorous acid with no additive), HP3-U (phosphorous acid with urea), P3-NWD (phosphite with no wet–dry cycles), P1-U (hypophosphite), and thio means (thiourea).

**Table 4:** ^31^P-NMR relative abundances ^1^ (%) of the organic P compounds detected in various reaction samples.

Sample Name	Phosphite (Unreacted)	Orthophosphate	5′-mono-PO_3_	5′-mono-PO_4_	2′-or 3′-mono-PO_3_	2′-or 3′-mono-PO_4_	Nucleoside Diphosphite Species	Glycerol-1-PO_3_	Glycerol-1-PO_4_	Glycerol-2-PO_3_	Glycerol-2-PO_4_	Total Org. PO_4_	Total Org. PO_3_	^T^C-O-P
	a	b	g	h	i	j	k	l	m	n	o			
A	93	3	3	BDL	1	BDL	BDL	----	----	----	----	BDL	4	4
B	1	BDL	44	BDL	20	BDL	35	----	----	----	----	BDL	99	99
C	88	6.5	----	----	----	----	----	3.5	BDL	2	BDL	BDL	5.5	5.5
D	78	2	----		----	----	----	10	6	3	1	7	13	20
E	98	1	1	BDL	BDL	BDL	----	----	----	----	----	BDL	1	1
F	44	0.5	36	1	18	0.5	----	----	----	----	----	1.5	54	55.5

^1^ The relative abundances (%) of the phosphonylated/phosphorylated products were calculated on the basis of the total P dissolved and by the peak integration method, as reported previously [24,25,26]; ^T^C-O-P means total C-O-P (carbon-oxygen-phosphorus) type organophosphorus compounds e.g., total sum of organic phosphates and phosphites for that particular reaction (and may not represent the exact sum due to rounding). The blank lines in the table show that these compounds are not present in the sample. BDL signifies below detection limit.

## Data Availability

NMR raw files and all the other relevant research results can be obtained by request from the corresponding author.

## Supplementary Materials

The following supporting information can be downloaded at: https://www.mdpi.com/article/10.3390/life13040920/s1, Figure S1: H-coupled ^31^P-NMR spectrum of Sample P3-NaCl; Figure S2: ^31^P-NMR (H-coupled) spectrum of sodium phosphite [Pi(III)] solution prior to reaction; Figure S3: H-coupled ^31^P-NMR of heating sodium hypophosphite and urea solution (mixture); Figure S4: ^31^P-NMR (H-coupled) spectrum of sodium hypophosphite [Pi(I)] solution prior to reaction showing no impurities or air oxidation phosphate species present in the starting compound; Figure S5: H-coupled ^31^P-NMR of phosphorylation and phosphonylation reactions of glycerol spiked with standard glycerol phosphate (isomeric) solution; Figure S6: H-coupled ^31^P-NMR of phosphorylation and phosphonylation reactions of adenosine spiked with standard 5’-AMP; Figure S7: H-coupled ^31^P-NMR spectrum of Sample P3-thio.
