# Supplementary material for: Prebiotic Chemistry of Phosphite: Mild Thermal Routes to Form Condensed-P Energy Currency Molecules Leading Up to the Formation of Organophosphorus Compounds
Source: Life (Basel). 2023 Mar 31;13(4):920. doi: 10.3390/life13040920 (PMC10144983; doi:10.3390/life13040920)
Supplement: Supplementary file 1 [file life-13-00920-s001.zip › life-2289988-supplementary.pdf]

Supplementary information

Prebiotic Chemistry of Phosphite: Mild thermal routes to form Condensed-P Energy Currency Molecules leading up to the formation of organophosphorus compounds

Maheen Gull[1]*, Tian Feng[1], Harold A. Cruz[2], Ramanarayanan Krishnamurthy[2] and Matthew A. Pasek[1]

[1]School of Geosciences, University of South Florida, Tampa, FL 33584, USA, [2]Department of Chemistry, The Scripps Research Institute, La Jolla, California, 92037 USA
Email: M.G; *ambermaheen@yahoo.com

Figure S1

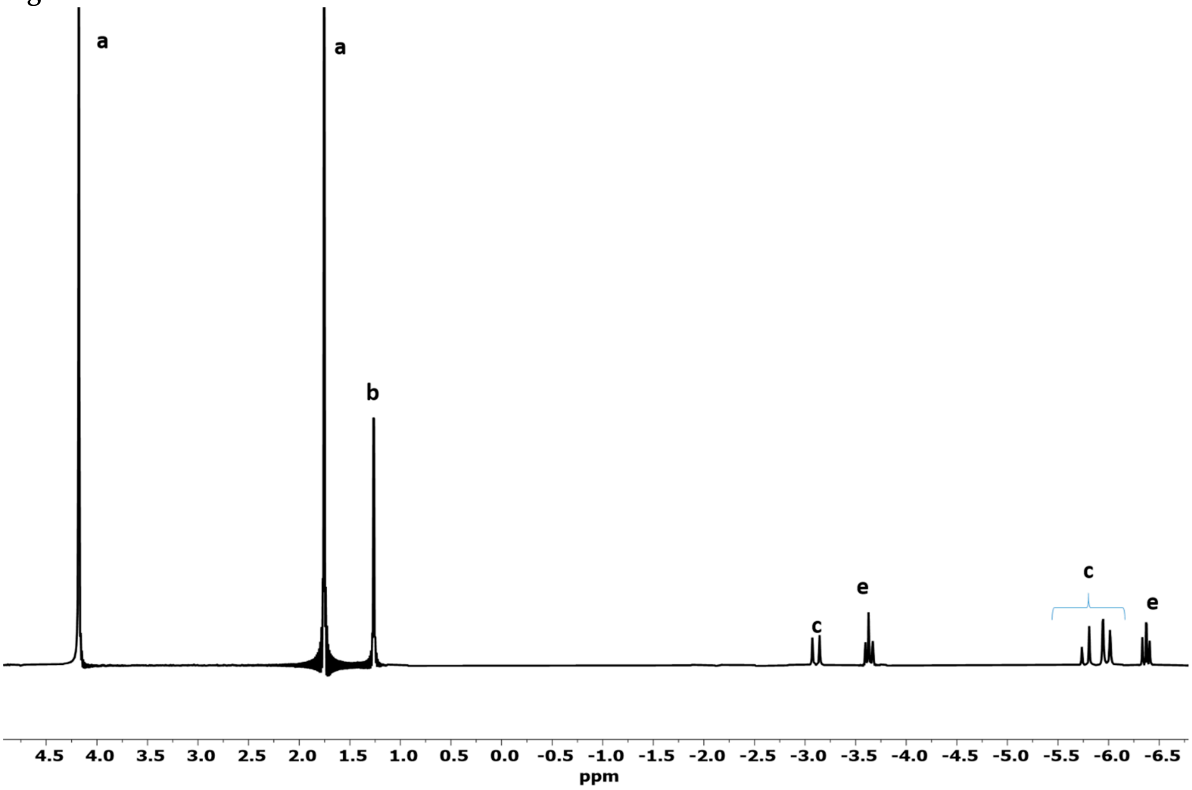

Figure S1: H-coupled ^{31}P-NMR spectrum of Sample P3-NaCl. Various inorganic P structures are as follows; (a) phosphite, (b) phosphate, (c) isohypophosphate, (e) pyrophosphite. Labeling is consistent with the text and the ^{31}P-NMR figures.

Spiking of the reaction samples with standard organic-P compounds

Standard included: uridine-5-monophosphate (5′-UMP) and adenosine-5-monophosphate (5′-AMP). The standard solutions were prepared by adding 0.05 g of the standard compound in 2mL DDI water. The mixture was stirred until completely dissolved and and about 10 μL was directly added into an NMR tube

containing reaction sample solution, followed by gentle shaking. The sample containing standard then was analyzed by ^{31}P-NMR, following the similar reaction protocol (similar no of scans) as for the reaction sample without spiking.

Figure S2

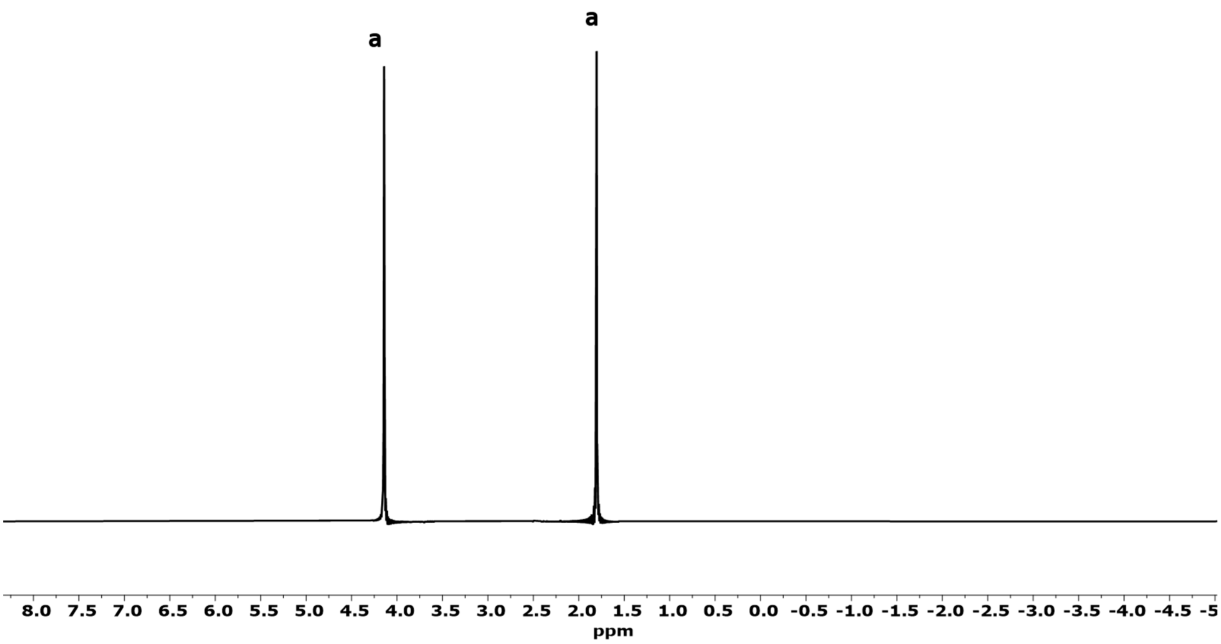

Figure S2 ^{31}P-NMR (H-coupled) spectrum of sodium phosphite [Pi(III)] solution prior to reaction showing no impurities or air oxidation phosphate species present in the starting compound.

Figure S3

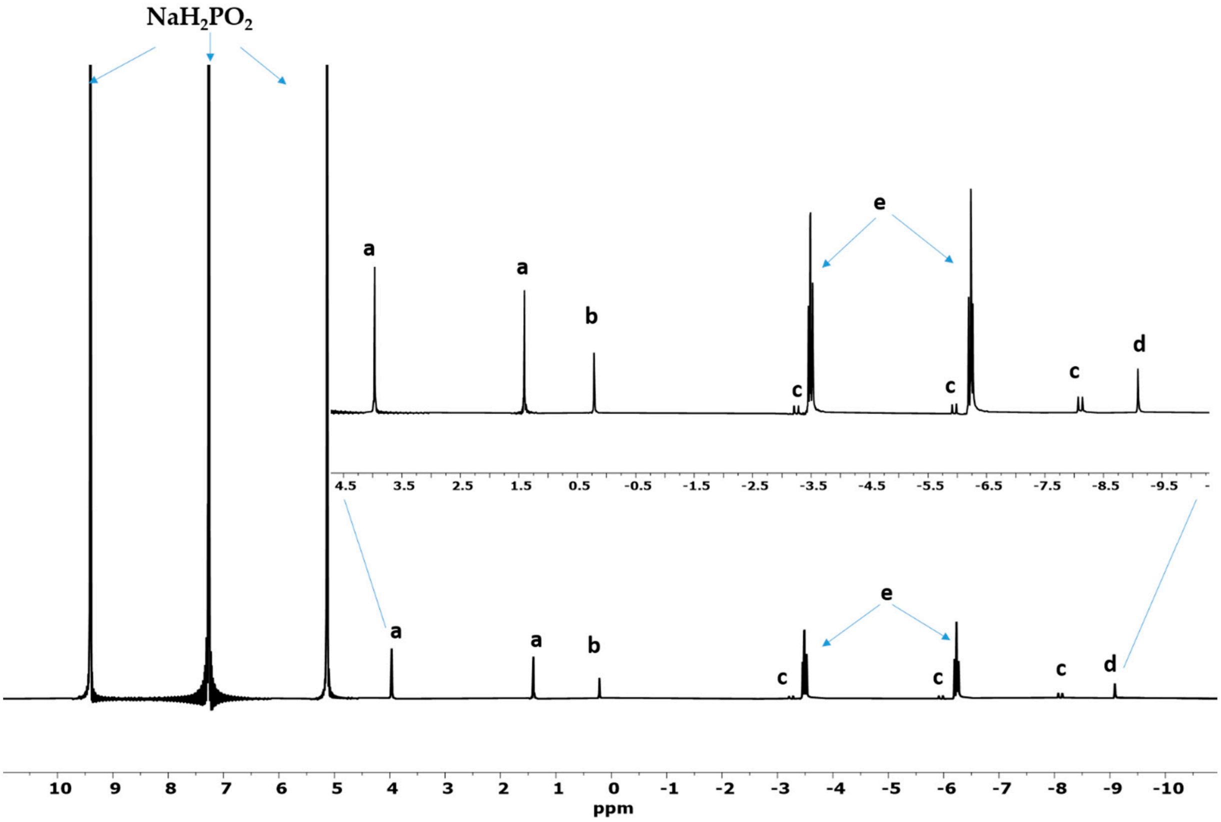

Figure S3 H-coupled ^{31}P-NMR of heating sodium hypophosphite and urea solution (mixture) Sample P1-U at 78-83 °C to dryness for 2 days. The heating and evaporation leading to dryness of the solution oxidized some of the hypophosphite [Pi(I)] into phosphite [Pi(III)], phosphate [Pi(V)], pyrophosphite [PPi(III)]., isohypophosphate [PPi(III-V)] and pyrophosphate [PPi(V)]. Peak labeling is consistent with all the other samples. The details of each peaks have been discussed in the text of the manuscript.

Figure S4

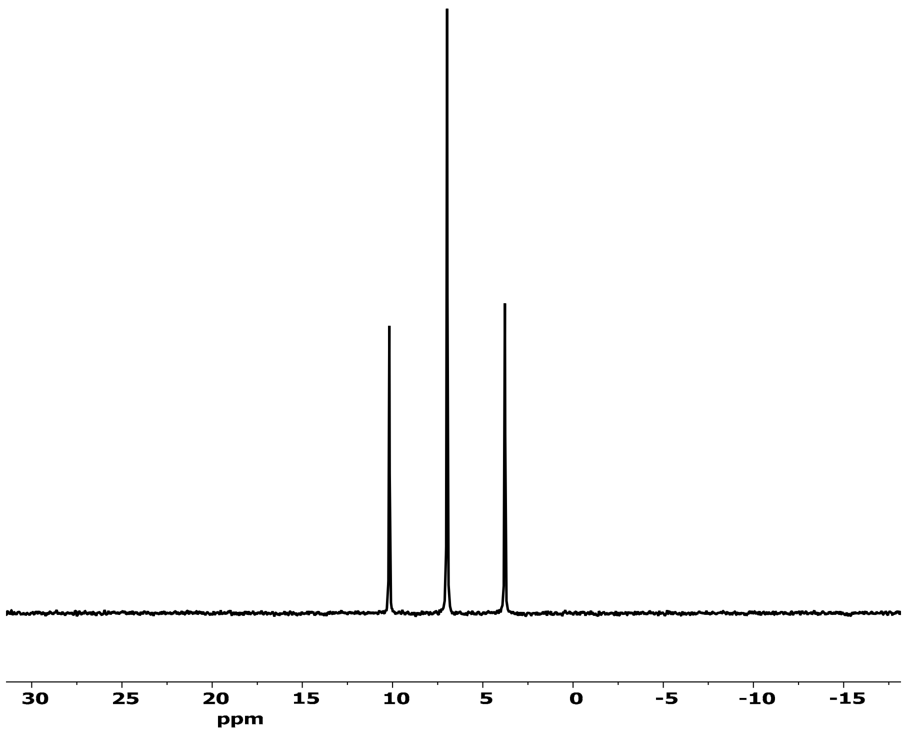

Figure S4 ^{31}P-NMR (H-coupled) spectrum of sodium hypophosphite [Pi(I)] solution prior to reaction showing no impurities or air oxidation phosphate species present in the starting compound.

Figure S5

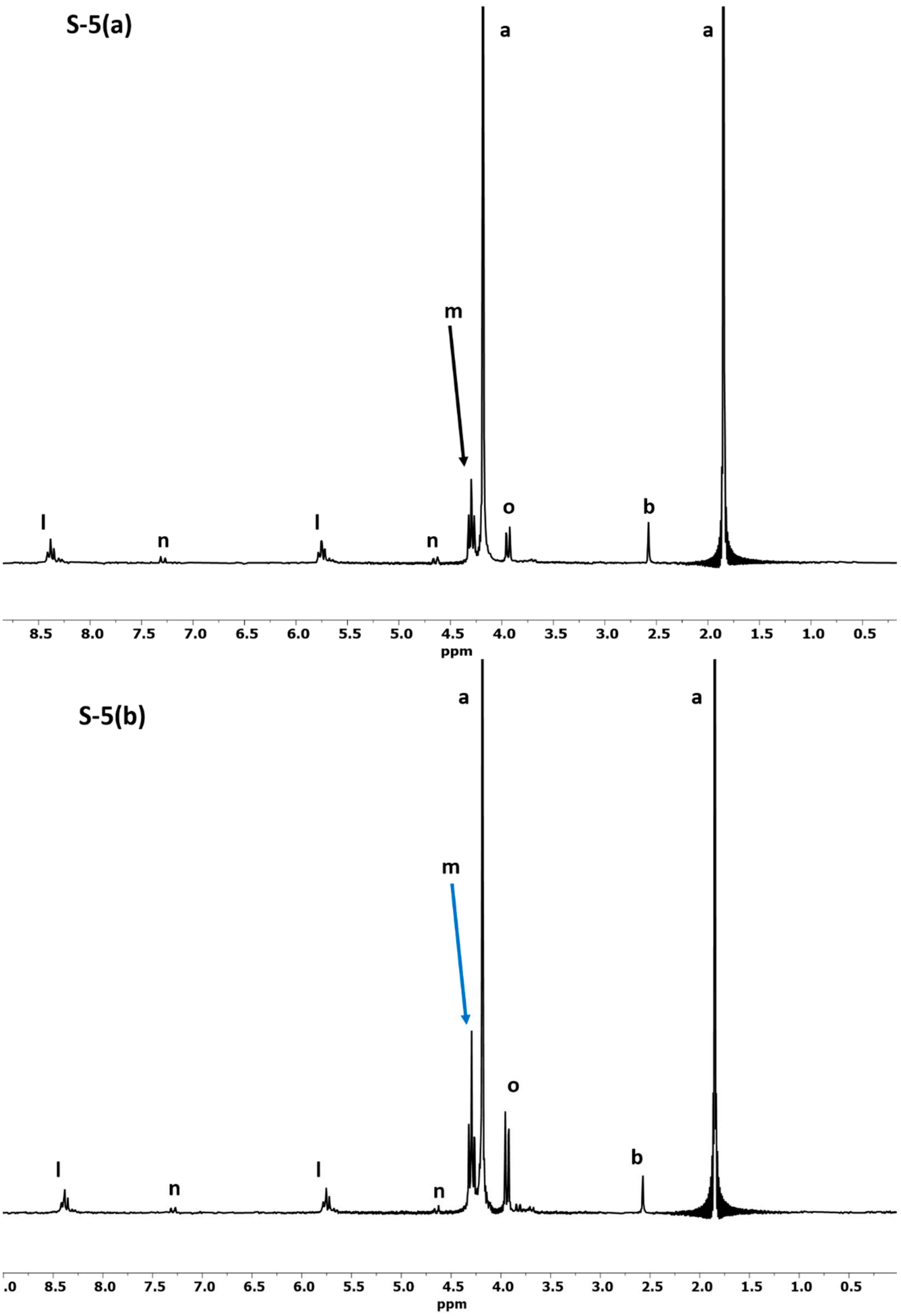

Figure S5 (a) H-coupled ^{31}P-NMR of phosphorylation and phosphonylation reactions of glycerol (sample D). The labeled peaks correspond to the following P compounds; (a) phosphite, (b) phosphate, (l) glycerol-1-phosphite, (m) glycerol-1-phosphate, (n) glycerol-2- phosphite, (o) glycerol-2-phosphate, respectively. The black arrow pointing peak

m which was identified and confirmed to be glycerol-1-phosphate after the same reaction sample was spiked with standard glycerol phosphate (isomeric mixture) solution and the location (ppm) of 'peak m' matched with that of the standard. Figure S5 (b) shows the H-coupled ^{31}P-NMR of the sample spiked with standard glycerol phosphate (isomeric) solution (10 μL). The blue arrow represents the spiked compound's location that exactly overlapped that of the triplet m, around 4.2 to 4.3 ppm. More broadening in the spiked sample peak m can be seen due to the overlapping of the standard compound and the reaction sample. Since standard solution was an isomeric mix. (e.g., containing both glycerol-1-phosphate and glycerol-2-phosphate), increase in the peak o height is justified by the presence of glycerol-2-phosphate exactly matched with the one present in the reaction sample.

Figure S6

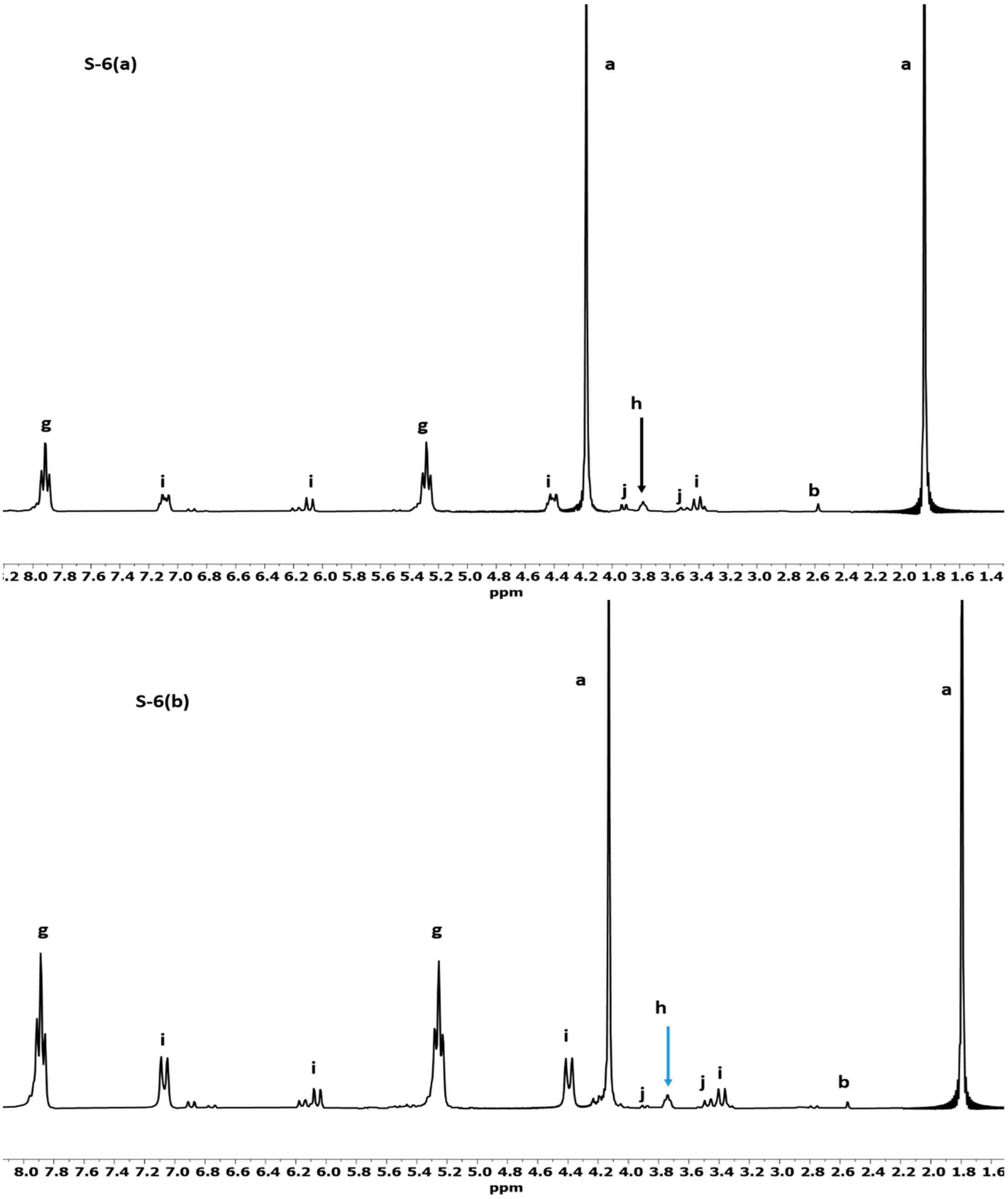

Figure S6 (a) H-coupled ^{31}P-NMR of phosphorylation and phosphonylation reactions of adenosine (sample F). The labeled peaks correspond to the following P compounds; (a) phosphite, (b) phosphate, (g) 5′-adenosine phosphite, (h) 5′-AMP, (i) 2′- and 3′-adenosine phosphites, (j) 2′- or 3′-AMP, respectively. The black arrow pointing peak h which was identified and confirmed to be 5′-AMP after the same reaction sample was spiked with standard 5′-AMP solution and the location (ppm) of 'peak h' matched with that of the standard. Figure S-6(b) shows the H-coupled ^{31}P-NMR of the sample spiked with standard 5′-AMP solution (10 μL). The blue arrow represents the spiked compound's location that exactly overlapped that of the triplet h, around 4.2 to 4.3 ppm.

Figure S7

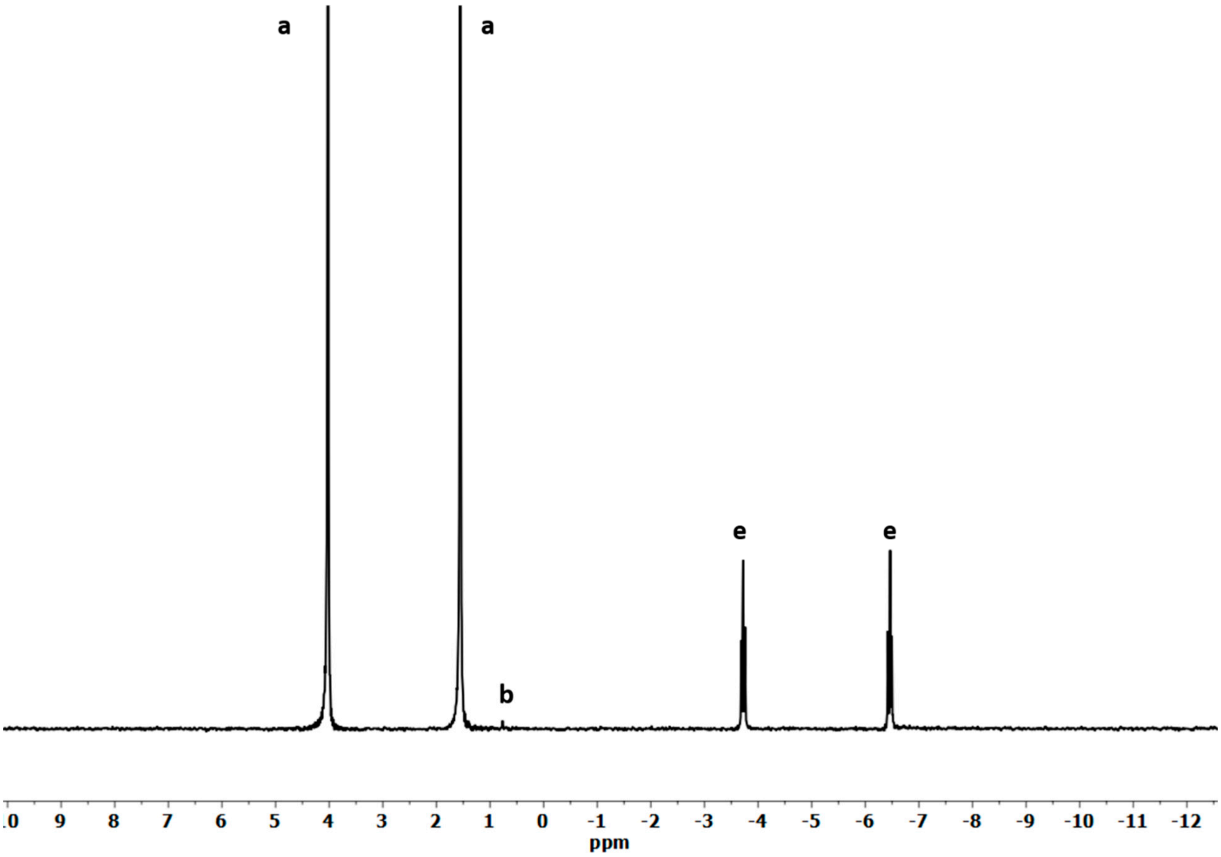

Figure S7: H-coupled ^{31}P-NMR spectrum of Sample P3-thio. Where (a) phosphite, (b) phosphate (almost negligible amount), (e) pyrophosphite, respectively.
